# Development and Application of a Novel QuEChERS Method for Monitoring of Tributyltin and Triphenyltin in Bottom Sediments of the Odra River Estuary, North Westernmost Part of Poland

**DOI:** 10.3390/molecules25030591

**Published:** 2020-01-29

**Authors:** Dawid Kucharski, Przemysław Drzewicz, Grzegorz Nałęcz-Jawecki, Kamila Mianowicz, Artur Skowronek, Joanna Giebułtowicz

**Affiliations:** 1 Department of Bioanalysis and Drugs Analysis, Faculty of Pharmacy, Medical University of Warsaw, ul. Banacha 1, 02-097 Warszawa, Poland; dkucharski@wum.edu.pl; 2 Polish Geological Institute-National Research Institute, ul. Rakowiecka 4, 00-975 Warszawa, Poland; asko@pgi.gov.pl; 3 Department of Environmental Health Sciences, Faculty of Pharmacy, Medical University of Warsaw, ul. Banacha 1, 02-007 Warszawa, Poland; gnalecz@wum.edu.pl; 4 Institute of Marine and Environmental Sciences, University of Szczecin, Wały Chrobrego 1-2, 70-500 Szczecin, Poland; Kamila.Mianowicz@usz.edu.pl

**Keywords:** tributyltin, triphenyltin, QuEChERS extraction, bottom sediments, Odra River estuary, LC-MS/MS

## Abstract

A Quick, Easy, Cheap, Effective, Rugged, and Safe (QuEChERS) extraction method combined with Liquid Chromatography-Tandem Mass Spectrometry (LC-MS/MS) for determination of organotin compounds (OTC) has been newly developed. The novel analytical method was validated and the quality of the results was tested by the use of certificate reference material of freshwater sediment BCR 646. The method was applied in determination of OTC concentration in real samples of bottom sediments collected from the Polish part of Odra River Estuary. The samples came from locations with different anthropogenic impact. Additionally, the extraction recovery of OTC and matrix effect on MS signal response was investigated based on those real environmental samples. It was found that organic compounds and anthropogenic contaminations present in bottom sediments may affect extraction efficiency of the organotin compounds (OTC) and change the matrix effect on MS signal response. The highest concentrations of tributyltin were found in bottom sediments collected from locations in vicinity of the Szczecin harbor and shipyards. The presence of triphenyltin above limit of detection (5 ng TPhT/g of sediment) was observed only in two samples and its concentration was several times lower compared to concentration of tributyltin (from 58 ng/g to 5263 ng/g). In spite of the fact that, the application of TBT-based paints on hull of vessel entering EU ports has been banned by European Commission regulation No. 782/2003 since 2008, the OTC compounds are still present in bottom sediment and pose significant threat to the environment. This threat should be taken into account during dredging of waterways and other hydrotechnical works.

## 1. Introduction

Organic tin compounds (OTCs) have been widely used in industry, mainly for plastic manufacturing and in agriculture for crop protection [1]. However, OTCs have been mostly added to antifouling paints for protection of surface of ship hull against growth of microorganisms, plants, algae, or molluscs [2]. Because of OTCs high toxicity to environment, the usage of them was banned by International Marine Organization since 1st January 2003. Despite that, OTCs are still present in environment, especially in vicinity of shipyards, harbors or waterways [3]. Among organic tin compounds tributyltin (TBT) and triphenyltin (TPhT) are the most toxic to organisms. Those compounds attenuate oxidative phosphorylation in mitochondria [4], disrupt steroid homeostasis [5] and inhibit gene expression [6]. It was also reported that low concentrations of TBT and TPhT effect on imposex (the development of male characteristic in females) in many aquatic organisms [7].

TBT and TPhT are slightly soluble in water and thus their concentration levels in surface waters reported in literature are very low [8,9,10]. These compounds tend to accumulate in bottom sediments due to the ion-exchange and hydrophobic interactions with clay minerals and organic matter, respectively [11]. However, they may be released from the sediments during various biological processes or even seafaring during summertime [12]. Organotin compounds are very persistent in bottom sediments and pose risk for environment for a long time [13].

The gas chromatography with various detectors such as mass spectrometry, flame photometric detector, atomic emission detector, or, recently introduced, inductively couple plasma mass spectrometry have been widely used for detecting of organotin compounds (Table A2). In recent years inductively coupled plasma atomic emission spectroscopy analytical technique is also gained importance [14]. The most crucial step of OTC determination in sediments is the compounds extraction. Different non-polar solvents such as dichloromethane, hexane and toluene are used in these procedures. In order to improve efficiency, hydrochloric acid or organic acids such as boric acid are added to sample [15]. Lowering of sample pH decrease dissociation of OTC cations and reduce negative charge sites on surface of clay minerals and organic matter (mostly humic acids) present in the sediments. Moreover, addition of carboxylic acids reduces decomposition of OTC during sonication or microwave assisted extraction of the sample at elevated pressure and temperature (free radical scavenging). Besides, a complexing agent, for example, tropolone is added in order to improve the efficiency [16]. Trialkyl tin compounds are usually derivatized in situ to volatile tetraalkyl compounds by sodium tetraethylborate (NaBEt_4_) prior GC analysis [17]. The major interference is elemental sulfur and sulfur containing compounds frequently present in the sediments [18]. Sulfur and sulfur compounds also undergo alkylation in reaction with NaBEt_4_ [19,20]. Those compounds interfere in analysis of organic tin compounds by gas chromatography with mass spectrometry detector. Therefore, desulfurization step including the use of activated copper, oxidation by dimethyldioxirane (DMD) following by absorption by Al_2_O_3_ [19] is added to analytical procedure. Sulfur interferences may be removed by application of microwave assisted extraction at elevated temperature and pressure [21]. In the case of analysis of sediments, it is necessary to add more reagents during derivatization reaction in order to compensate for the consumption of reagents by side reactions with metal cations and other constituents in the matrices [22]. However, derivatisation step is not required for liquid chromatography (LC) allowing faster analytical procedures and eliminating a potential source of cross-contamination [13].

Extraction method introduced by Anastassiades [23] in 2002 and later validated by Lehotay [24] is one of the most promising user-friendly and high throughput extraction procedure that following green chemistry principles, involving reduced sample and organic solvents amounts used in the analysis. The method based on acetonitrile extraction/ partitioning of pesticides residues that covers a broad type of analytes, ranging from non-polar to very polar compounds. The method called QuEChERS from the Quick, Easy, Cheap, Effective, Rugged, and Safe is an effective sample preparation approach based on partitioning via salting-out extraction where an equilibrium between an aqueous and an organic layer (acetonitrile—ACN) is established [25]. The next step is dispersive solid-phase extraction step that involves further clean-up using several combinations of porous sorbents and salts to remove matrix interfering substances. QuEChERS approach was quickly found application in analysis of many other compounds in various biological and environmental matrices [25,26]. Nowadays, the method is adopted according to target analyte properties, matrix composition, equipment, and analytical techniques available in the laboratory.

The QuEChERS approach has been already used to extract tributyltin from *Cunninghamella echinulate* cultures [27]; however, the method was not validated. Despite of numerous analytical methods used for determination of organotin compounds in sediments [13,28], the QuEChERS extraction combined with liquid chromatography mass spectrometry (LC-MS) for determination of tributyltin (TBT) and triphenyltin (TPhT) in bottom sediments was not developed and validate so far. Therefore, the aim of this work was to devise, develop and validate a QuEChERS sample preparation method for determination TBT and TPhT in bottom sediments from the Odra River Estuary (north-westernmost part of Poland) by the means of LC-MS/MS. The amount and combination of solvents and salts were optimized in terms of highest peak intensity of TBT and TPhT in extract from the sediments. The effect of sediments constituents on the ionization suppression or enhancement and extraction efficiency was also discussed. The optimized parameters of the method were tested and validated by the use of certificated reference material BCR646.

## 2. Results and Discussion

### 2.1. Optimisation of the QuEChERS Extraction Procedure

The method optimization was performed and following parameters were established: Effect of composition of extracting solutions, QuEChERS salts composition, d-SPE clean-up, extraction time, and agitation technique.

#### 2.1.1. Effect of Composition of Solutions and QuEChERS Salts

The water and organic phase composition as well as salt addition for salting-out the analyte were optimized. The best salting-out of ACN is usually achieved by addition MgSO_4_ whereas addition of NaCl controls polarity of extraction solvents and thus allows to control the selectivity of the extraction [26]. However, the use of these salts can decrease detection limit and peaks intensity due to deposition of solid NaCl in MS source [29]. Therefore, the effect following three different QuEChERS salt compositions on extraction efficiency were investigated: 1) As was reported in the CEN Standard Method EN 15662 ([30]: 100 mg MgSO_4_, 25 mg NaCl, 25 mg Na_3_Citrate and 12.5 mg Na_2_Citrate·H_2_O; 2) as was reported in AOAC Official Method [31]: 100 mg MgSO_4_ and 40 mg sodium acetate (NaOAc) and 3) 100 mg of ammonium acetate (NH_4_OAc). The effect of solvent composition on extraction efficiency was tested: MiliQ water, solution of 30 mM potassium phosphate monobasic solution in MiliQ water (pH = 7) as an aqueous phase and, as an organic phase, acetonitrile and solution of 5% formic acid in acetonitrile. Tributyltin and triphenyltin extractions were performed in all combinations of solvents with the use of three sets of QuEChERS salts (CEN Standard Method EN 15662, AOAC Official Method and NH_4_OAc).

**Organic solution:** The results showed that the use of formic acid (5% in ACN) greatly increases peak intensity of TBT and TPhT. This is a result of adjustment of solution pH to around 5. At pH below pKa = 6.25, tributyltin is predominantly present in cationic form TBT+; thus, desorption from sediments is favoured [32,33]. The same is for TPhT, which pKa = 5.2 [32]. Moreover, surface of clay minerals is positively charged and humic acids are protonated in such conditions [32]. Therefore, there are no negative charge sites on surface sediment that may bond organotin cations.

**Aqueous solution:** 30 mM potassium phosphate monobasic solution (pH = 7) as aqueous phase was used as well. However, it was not observed any extraction efficiency improvement; thus for further analysis MiliQ water was used.

**Figure 1:**
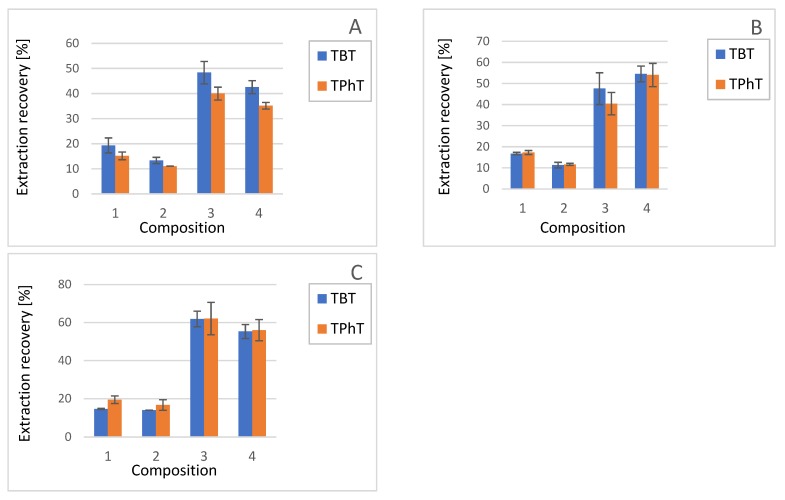
Extraction recovery of tributyltin (blue column) and triphenyltin (orange column) with the use of three QuEChERS salts **A**) 100 mg MgSO_4_, 25 mg NaCl, 25 mg Na_3_Citrate, and 12.5 mg Na_2_Citrate·H_2_O; **B**) 100 mg MgSO_4_ and 40 mg NaOAc and **C**) 100 mg NH_4_OAc as QuEChERS salts. As an aqueous phase MiliQ water (composition 1 and 3) or monopotassium phosphate solution (composition 2 and 4) was used. As an organic phase pure acetonitrile (composition 1 and 2) or 5% formic acid in acetonitrile solution (composition 3 and 4) was used.

**QuEChERS salts**: The highest recoveries, both for TBT and TPhT, were observed in the presence of ammonium acetate. Contrary to sodium carboxylate salts, ammonium acetate salts easily vaporize and do not form solid deposits in electrospray ionisation (ESI) source of MS. Moreover, ammonium salts sometimes enhance ionization of analytes [34]. Therefore, ammonium acetate was used for further optimization of QuEChERS extraction procedure. The use of NH_4_OAc, 200 µL MiliQ water and 5% formic acid in acetonitrile resulted in the highest extraction recovery for TBT 63.5% (SD = 7.5%, *n* = 5) and 62.4% for TPhT (SD = 8.5%, *n* = 5). The summary of the results of the investigations on the effect of various compositions of extracting solutions (Table 1) and composition of QuEChERS salts is presented in Figure 1.

#### 2.1.2. Effect of d-SPE Clean-Up

**Figure 2:**
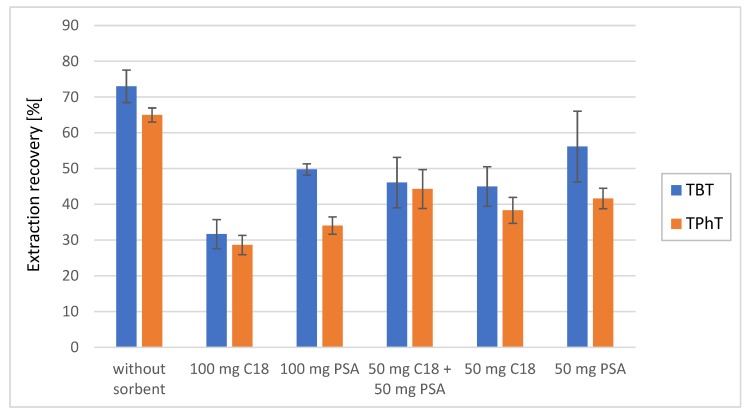
Extraction recovery of tributyltin (blue column) and triphenyltin (orange column) from sediments depending on using of different sorbents: C18 (octadecyl bound silica) and PSA (primary–secondary amine bound silica). As aqueous phase MiliQ water and as an organic phase 5% formic acid in acetonitrile solution were used with ammonium buffer as QuEChERS salt.

High amounts of organic compounds in bottom sediments may cause faster stationary phase wearing off, sometimes suppress MS signal and affect validation parameters i.e.; lower limit of quantification (LLOQ), accuracy, and precision. Therefore, clean-up organic extract of the sediments before LC-MS/MS analyses is often recommended. In QuEChERS method acetonitrile extract of sample is clean-up by dispersive solid phase extraction (d-SPE). In d-SPE clean-up, octadecylsilane (C18) or primary–secondary amine (PSA) sorbents are frequently used for matrix interferences removal and for improving efficiency of chromatographic separation and MS signal performance [35]. In this study the application of PSA and C18 for clean-up of sediments extract did not bring any improvement in LC-MS analysis of TBT and TPhT. Extraction efficiency without clean-up was 73.1% (SD = 4.9%, *n* = 5) for TBT and 65.1% (SD = 2.0%, *n* = 5) for TPhT whereas, in the case of application of 100 mg C18, the efficiency dropped to 32.2% (SD = 4.1%, *n* = 5) for TBT and 29.8% (SD = 2.7%, *n* = 5) for TPhT (Figure 2). Similar recoveries were obtained when 100 mg PSA, 50 mg C18, and their combination 50 mg C18 and 50 mg PSA was applied. It suggests that TBT and TPhT retained on PSA and C18 sorbents also as a complex with organic components of the matrix. Further study with other sorbent might be considered. However, in this case, additional clean-up step was not necessary. Clean-up by d-SPE did not improve performance of chromatographic separation and peak shape. No improvement of MS signal response, i.e.; signal to noise ratio and signal stability, was observed, even after more than 100 injections of samples with the same concentration of TBT and TPhT in the sediment. Hence, in further analysis of TBT and TPhT in sediments, the step of extract clean-up was excluded from the analytical method. As a result, the procedure of samples preparation became less laborious and time consuming.

#### 2.1.3. Effect of Extraction Time and Agitation Technique

**Figure 3:**
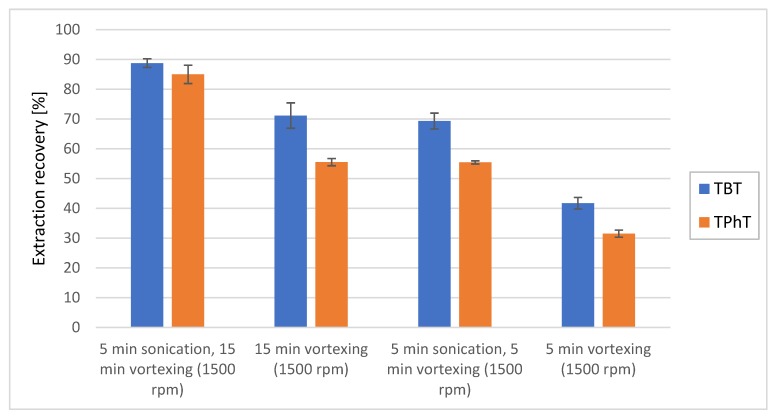
Extraction recovery of tributyltin (blue column) and triphenyltin (yellow column) from sediments depending on different extraction techniques and durations. As aqueous phase MiliQ water and as an organic phase 5% formic acid in acetonitrile solution were used with ammonium buffer as QuEChERS salt.

The time and simplicity of analysis are key factors in daily application of analytical methods. Therefore, extraction time and agitation technique in terms of extraction efficiency were optimized. The agitation of sample during extraction was carried out by vortexing with or without following sonication. The results show that the use of sonication greatly improve extraction efficiency what was reported previously [36]. Extraction efficiencies after 5 min of vortexing were 42.5% (SD = 1.9%, *n* = 5) for TBT and 32.6% (SD = 1.2%, *n* = 5) for TPhT whereas additional 5 min of sonication increased efficiencies to 69.3% (SD = 2.7%, *n* = 5) for TBT and 55.7% (SD = 0.5%, *n* = 5) for TPhT (Figure 3). Additionally, peaks intensities for 5 min of sonication and 5 min of vortexing were not statistically different in comparison to 15 min of vortexing alone (*p* = 0.2838 for TBT and *p* = 0.2021 for TPhT). Therefore, application of combined vortexing and sonication may reduce extraction time significantly. It is worth to mention that the highest extraction efficiencies were obtained after 15 min of intensive vortexing following by additional sonication for 5 min. The obtained extraction efficiency were 89.0% (SD = 1.5%, *n* = 5) for TBT and 85.6% (SD = 3.1%, *n* = 5) for TPhT. However, extraction efficiency obtained after 5 min of vortexing and sonication is good compromise between desired duration of the extraction procedure and obtained extraction efficiency of organotin compounds.

### 2.2. Method Validation

The method validation was performed and following parameters were established: analytical range (linearity), accuracy, precision, stability, selectivity and carry-over, matrix-effect, recovery according to EMA Guideline for Process Validation. Validation was carried out on sediments collected from the Odra River Estuary.

#### 2.2.1. Lower Limit of Quantification and Limit of Detection

LLOQ defined as the lowest calibration standard on the calibration curve detected with appropriate precision and accuracy was 1 ng/g and 5 ng/g for TBT and TPhT respectively. LOD defined as the lowest analyte concentration that can be reliably detected was 0.6 ng/g and 2.4 ng/g for TBT and TPhT respectively.

#### 2.2.2. Analytical Range

Analytical range (linearity) is the range where MS signal is proportional to analyte concentration in a sample without dilution. The response of MS instrument was tested for seven concentrations of the organotin compounds in MiliQ water (*n* = 6). The calibration curve obtained by application of weighted linear regression (*1/x*) was linear in the range 1–4000 ng/g for TBT and 5–4000 ng/g for TPhT. The values of regression parameters for the curve, described by the equation: y = ax + b, were calculated as: a = 0.00073 (SD = 0.00021, *n* = 6), b = 0.0052 (SD = 0.0121, *n* = 6) and R^2^ = 0.992 for TBT and a = 0.00157 (SD = 0.00005, *n* = 6), b = −0.012 (SD = 0.028, *n* = 6), and R^2^ = 0.994 for TPhT. All regression parameters were statistically significant (*p* < 0.05).

#### 2.2.3. Accuracy and Precision

**Figure A1:**
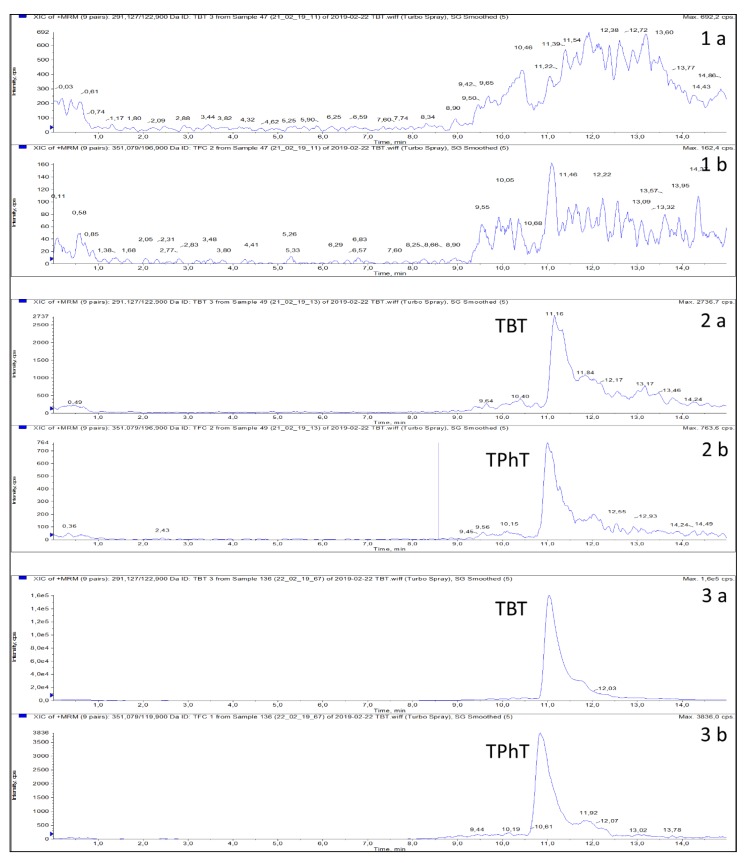
Chromatograms of TBT and TPhT in blank sample (1a for TBT and 1b for TPhT), lower limit of quantification (LLOQ) (2a for TBT and 2b for TPhT) and real sample (3a for TBT and 3b for TPhT).

Accuracy and precisions are two the most important elements of a chromatographic analytical method. Accuracy is defined as a measure of closeness of the experimental result of analyte determination to the real amount of the substance in the real matrix. The precision of a method is a measure of how variable are the experimental results when the method were applied under well-controlled conditions [37]. In the validation process the accuracy and precision for LLOQ and QC samples within one day (*n* = 5) and between runs (*n* = 15) met the acceptance criteria of Medicines Agency Guideline for Process Validation (Table 2). Chromatograms of control samples of sediments, LLOQ and selected sediment sample are presented in Appendix (Figure A1). The signal to noise ratio for LLOQ was 5.3 and 7.2 for TBT and TPhT, respectively.

#### 2.2.4. Selectivity and Carry-Over

Selectivity is a measure of the extent to which the method can determine a particular analyte in the matrix without any interference from matrix components. It was established as a ratio of peak area in the blank sample of bottom sediment (without OTC compound) at retention time corresponding to retention time of the analyte to peak area corresponding to concentration of lower limit of analyte quantification. Established selectivity was 6.1% (SD = 3.3%, *n* = 6) for tributyltin, 3.8% (SD = 2.6%, *n* = 6) for triphenyltin and 1.2% (SD = 0.2%, *n* = 6) for tributyltin d–27. Carry-over caused by contamination from preceding analyses is major problem that may affect the accuracy and precision of the analytical method. It was established as a ratio of peak area corresponding to the analyte concentration in the blank sample (A_blank sample_) to the peak area corresponding to concentration of lower limit of analyte quantification (A_LLOQ_) (Equation (1))

(1) $$Carry-over\;=\;[A_{blank\;sample}/A_{LLOQ}]\;\times 100\%,$$

The calculated carry-over was 1.21% (SD = 0.35%, *n* = 6) for tributyltin, 1.14% (SD = 0,53%, *n* = 6) for triphenyltin and 0.3% (SD = 0.1%, *n* = 6) for tributyltin d–27. All results met acceptance criteria (<20% for analyte and <5% for internal standard) of EMA Guideline for Process Validation.

#### 2.2.5. Stability

Stability is usually described as the degree of decomposition of analyte in matrix and stock solution under specific storage conditions after certain time. Stability of the analyte affects the trueness and precision of the analytical procedure. Stability of analytes was determined as a comparison of concentration of fortified blank samples of sediment to reference sample. The freeze/thaw stability was investigated in fortified sediments after 3 cycles of freezing and thawing. Short-term stability was established after 4 h storage of the solution at room temperature. Long term stability was established after 30 days storage of working solutions in −25 °C. Stability in the LC autosampler was established after 24 h and 48 h. The stability tests were repeated 5 times. Stability of stock solutions (10 µg/mL) after 30 days storage in −25 °C was 106% for TBT, 113% for TPhT and 85% for TBT-d27. All results met the acceptance criteria in range 85–115% of EMA Guideline for Process Validation (Table 3).

#### 2.2.6. Estimation of Trueness

Trueness is the closeness of agreement between a test result and the accepted reference value of the property being measured. Trueness is stated quantitatively in terms of “bias”, with smaller bias indicating greater trueness. Bias is typically determined by comparing the response of the method to a reference material with the known value assigned to the material [38]. Estimation of trueness was carried out by the use of certificate reference material (CRM) of freshwater sediments BCR^®^ 646; the certificated concentration of TBT and TPhT was 480 n/g and 29 ng/g. CRMs are traceable to international standards with a known uncertainty; thus, it can be used to assess simultaneously laboratory and method bias, assuming that there is no matrix mismatch [39,40]. Significance testing of the bias that took into account uncertainty of certificated value was performed. According to ISO Guide 33 [41], the expend uncertainty of the difference between certificated and measured value *U*_Δ_ with coverage factor k = 2, corresponding to a level of confidence 95%, is obtained by (Equation (2)):

(2) $$U_{\Delta }\;=\;k\cdot \sqrt{u_{ref}^{2}+\frac{S_{m}^{2}}{n}},$$

where: *u_ref_* is the uncertainty of the reference value taken from the certificate (40 n/g for TBT and 5.5 ng/g for TPhT); *s_m_* is a standard deviation calculated from the measured values; *n* is a number of repeated measurements, (*n* = 6).

The difference between the certified reference value (taken from the certificate −480 n/g for TBT and 29 ng/g for TPhT) and the mean measured value obtained by combined QuEChERS and LC–MS methods was Δ_TBT_ = 30 ng/g for TBT and Δ_TPhT_ = 1 ng/g for TPhT. The difference is within *U*_Δ_, thus the measured concentrations is compatible with reference concentrations.

#### 2.2.7. The Matrix Effect

Environmental matrix components often presented in non-clean-up extracts may affect ionization of analyte in MS. However there are many publications the purification step is not needed in extraction procedure [42]. Moreover, in this study, the results of method optimization have shown that commonly used sorbents for clean-up were not suitable for extract containing TBT and TPhT. Thus, it is very important to evaluate the matrix effect on signal response of the analyte. The presence of matrix effect results in poor analytical accuracy, linearity, and reproducibility, however, in the case of MS detector, selection of appropriate isotope labelled internal standard may control it [43]. In the study, deuterated TBT (TBT-d27) was used as an internal standard. Matrix effect was investigated in 10 samples of bottom sediments collected from the Odra River Estuary which were sieved to grain size below 0.6 mm. Detailed characteristics of the sediments are presented in Appendix (Table A1).

The absolute matrix effect (ME_A_) is described as an increase or decrease of MS signal response of analyte in the presence of environmental matrix in relation to the response of the same concentration of analyte in pure water solution (without environmental matrix). ME_A_ was in range 58–92% for TBT ($\bar{x}$ = 76.1%, SD = 9.4%) and 58–92% ($\bar{x}$ = 72.3%, SD = 10.4%) for TPhT. The results of Spearman′s correlation analysis suggest that compounds presents in anthropogenically impacted bottom sediments and granulometry may suppress the ionization of TBT in MS. Positive correlation was observed for polycyclic aromatic hydrocarbons (PAH) (r = 0.6930, *p* = 0.0263), heavy metals content (r = 0.6846, *p* = 0.0288), silt fraction (r = 0.7599, *p* = 0.1076) and negative correlation for sand fraction (r = − 0.7454, *p* = 0.0133). In the case of TPhT, the ionization suppression was positively correlated with content of total organic carbon (r = 0.6626, *p* = 0.0368), acid volatile sulfur (r = 0.7112, *p* = 0.0211), PAH (r = 0.8262, *p* = 0.0032) and silt fraction (r = 0.6524, *p* = 0.0409), and negatively correlated with sand fraction (r = −0.6443, *p* = 0.0443). It confirms that anthropogenic contaminations result in ionization suppression during MS analysis of organotin compounds. Other correlations were statistically insignificant.

**Figure 4:**
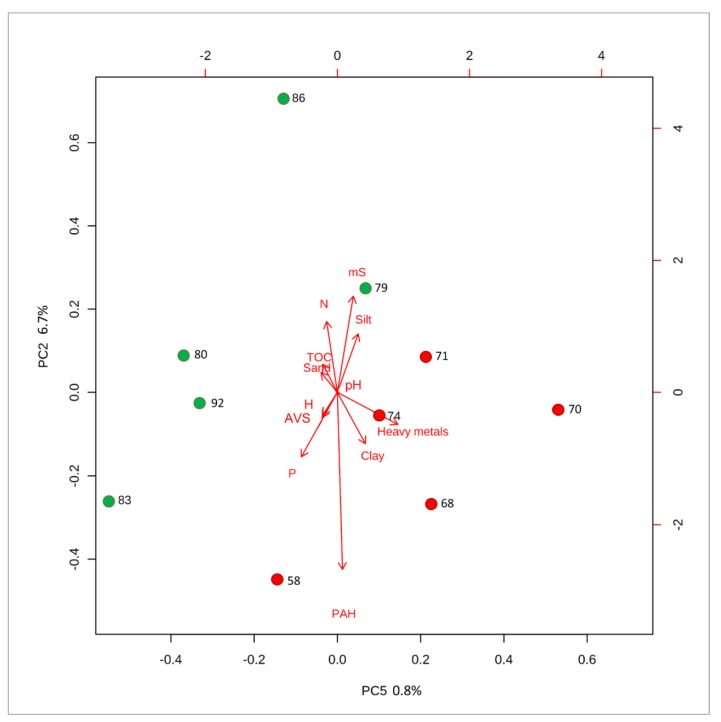
Principal component analysis (PCA)—distribution of the ME_A_ of TBT for a different sediments on a biplot (principal component 2 vs principal component 4). The sample labels refer to ME_A_ value. The biplot arrows point in the direction of increasing values for that variable. The length of the arrows approximates the variance of the variables, whereas the angles between them- approximate their intercorrelations. The variables are pH value, conductivity (mS), total organic carbon (%), total nitrogen content (%), total hydrogen content (%), acid volatile sulfur (%), total phosphorus content (%), sum of heavy metals (mg/kg), polycyclic aromatic hydrocarbons (µg/kg), clay content (%), silt content (%), and sand content (%).

**Figure 5:**
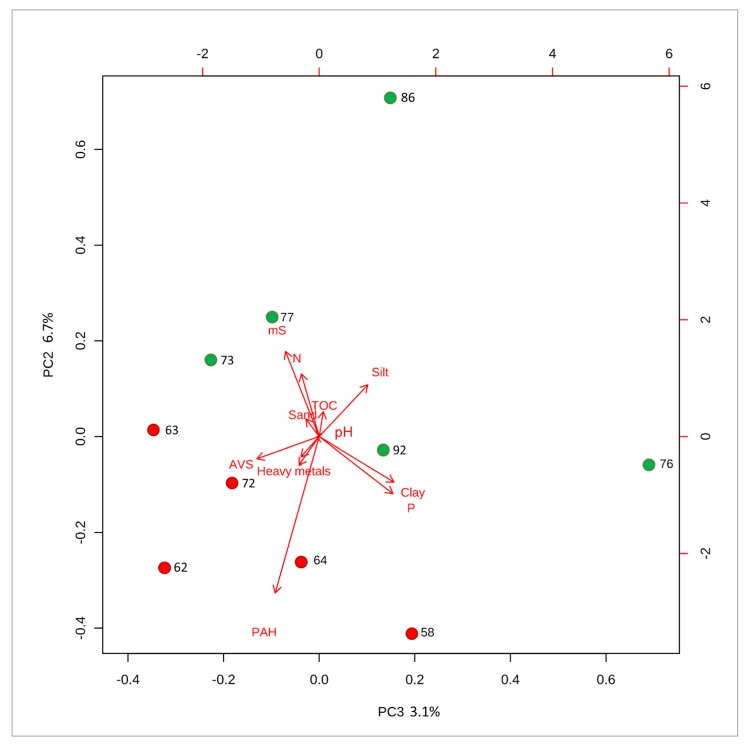
Principal component analysis (PCA)—distribution of the ME_A_ of TPhT for a different sediments on a biplot (principal component 3 vs principal component 4). The sample names refers to ME_A_. The biplot arrows point in the direction of increasing values for that variable. The length of the arrows approximates the variance of the variables, whereas the angles between them-approximate their correlations with pH value, conductivity (mS), total organic carbon (%), total nitrogen content (%), total hydrogen content (%), acid volatile sulfur (%), total phosphorus content (%), sum of heavy metals (mg/kg), polycyclic aromatic hydrocarbons (µg/kg), clay content (%), silt content (%), and sand content (%).

In order to better visualize all results and evaluate the relationships between the values of matrix effect and parameters presented in Table A1, principal component analysis (PCA) was performed (Figure 4 and Figure 5). PCA reduces the large number of parameters to interrelated variables and enabled to present data variation in a new coordinate system. The combination of principal components that adequately demonstrates differences between them was presented. Samples with the greatest ME_A_ value were marked by green dot. In the case of tributyltin (Figure 4), high content of heavy metals and clay fraction as well as low total organic carbon content, nitrogen content and sand fraction was associated with higher analytical signal suppression. For triphenyltin (Figure 5), high content of polycyclic aromatic hydrocarbons and slightly acid volatile sulfur content affected the matrix effect. It was also observed impact of silt fraction on decrease of analytical signal suppression. The results of PCA likely indicated that presence of petroleum oil compounds may affect nebulization and ionization of organotin compounds in ESI source of the mass spectrometer. For example, in the sample of sediment collected from port of Szczecin the concentration of PAH was 9828 mg/kg that resulted in ME_A_ = 58% (Table A1). Such high concentration of PAH indicates high pollution of oil compounds. Additionally, formation of charge neutral cluster or ion-pair with high molecular weight aromatic compounds or/and containing heteroatoms (nitrogen) is a plausible explanation of high ME_A _for this sample. Therefore, improvement of versatility of the analytical method will be a subject of further investigation.

Additionally, the mineral content of the bottom sediments is also important in MS determination of organotin compounds. Minerals may facilitate adsorption of organic compounds due to hydrophobic or/and ionic interactions. Especially, clay minerals (e.g.; kaolinite, montmorillonite) due to cationic exchange properties (at pH around 7) may adsorb not only organotin cations but also other compounds, especially containing nitrogen (amines, polycyclic aromatic nitrogen containing hydrocarbons and alkyl pyridinium surfactants). The clay minerals are predominant in fraction below 0.063 mm. The absolute matrix effect was established for 5 different samples of bottom sediments before and after sieving through 0.063 mm sieve. Decrease of ME_A_ level was observed both for tributyltin and triphenyltin in the samples of sediments after sieving. In the case of TBT, ME_A_ level decreased from 77.2 ± 7.5% to 65.6 ± 2.2% (*p* = 0.0389) and from 74.4 ± 7.1% to 65.6 ± 1.5% for TPhT (*p* = 0.0103). Although the amount of clay mineral fraction in bottom sediments did not have any direct effect on LC–MS method, the amount of interfering compounds increase with the increase of this fraction. This should be taken into account when the QuEChERS extraction method will be tailored to the analysis of bottom sediments from other part of world.

The absence of the ME_A_ is only desirable because suppression or enhancement of the signal does not influence quantification. But, the absence of the relative matrix effect (ME_R_) is crucial to obtaining reliable results and is mandatory during method validation. In the study the relative matrix were 4% for tributyltin and 5% for triphenyltin and met acceptance criteria (<15%). Although it was observed suppression or enhancement of the analytical signal for the individual sediment, the results of relative matrix effect show that the method can be considered as fully reproducible for analysis of TBT and TPhT in bottom sediments.

#### 2.2.8. Extraction Recovery

The extraction recovery for tributyltin was 85–106% ($\bar{x}$ = 93.2%, SD = 6.1%). Thus, almost all TBT was recovered from sediments. It was observed positive correlation with sand (r = 0.7356, *p* = 0.0153) and negative with silt fraction (r = −0.7256, *p* = 0.0175). The extraction recovery for TPhT was 85−99% ($\bar{x}$ = 90.5%, SD = 4.2%). Thus, almost all TPhT was recovered from sediments. RE was positively correlated with sand (r = 0.7112, *p* = 0.0211) and negatively correlated with PAH content (r = −0.7896, *p* = 0.0066), and silt fraction (r = −0.7226, *p* = 0.0182). The further statistical analyses were not conducted due to satisfactory efficiency of the validated method.

### 2.3. Application of the Method to the Real Environmental Samples

The earlier studies have shown the presence of elevate concentration of the organotin compounds, especially tributyltin and triphenyltin, in bottom sediments from the southern coast of the Baltic Sea. The concentrations detected in sediments collected from the Gdansk Gulf ranged from 2 ng/TBT/ g of sediment to almost 38,900 ng/TBT/g of sediment collected nearby Gdansk Shipyard. In comparison, in this study, the concentrations of TBT in sediments from the Szczecin Lagoon were from 5 ng/g to 280 ng/g. In the case of TPhT the highest concentration of triphenyltin was 961 ng/g of sediment from Gdansk shipyard [44], whereas in this study the concentrations of the compound determined in the most samples collected from the Odra River Estuary were below limit of detection.

**Figure 6:**
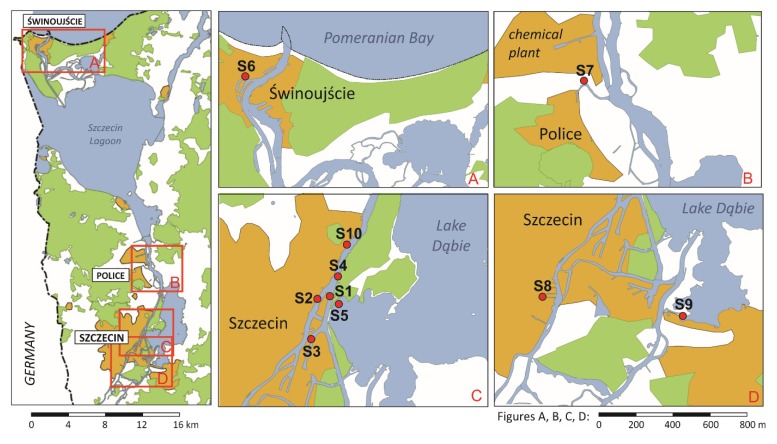
The map of points where samples of sediments were collected in the area of Świna River Estuary (**A**) and Larpia River-Police (**B**), Szczecin Harbor and Lake Dąbie (**C** and **D**). Geographic coordinates of sampling locations were given in Table 6.

In present study, developed combined QuEChERS and LC–MS method was applied to analysis of TBT and TPhT in below 0.063 mm fraction of bottom sediments from the Odra River Estuary (Figure 6). High concentration of TOC suggests that the sediments may accumulate large amounts of organotin compounds. High concentration of heavy metals found in those samples indicated that Odra River Estuary have been strongly impacted by industrial activity.

The highest concentrations of TBT were found in bottom sediment from the region of West Odra River (5263 ng/g), Gunica River (3884 ng/g), and Szczecin Shipyard (3296 ng/g) (Table 4). These are highly populated areas with developed maritime and shipyard industry. It corroborates the results of preceding studies that linked high concentration of TBT with maritime industry activity [44]. In the case of TPhT, the concentrations above LLOQ were detected only in bottom sediments collected nearby grain elevator “Ewa”, grain port quay (90 ng/g) and West Odra River (9 ng/g). TPhT was used as a fungicide in crop protection till 2002 (it was phase-out by Commission Decision of 20 June 2002, 2002/479/EC).

## 3. Materials and Methods

### 3.1. Reagents

Pure standards of tributyltin chloride TBT (96%), triphenyltin chloride TPhT (95%) and internal standard deuterated tributyltin chloride–d27 TBT-d27 (96%) were purchased from Sigma Aldrich (St. Louis, MO, USA). HPLC gradient–grade methanol, acetonitrile and formic acid 98% were purchased from Merck (Darmstadt, Germany). Quechers salts: Magnesium sulphate, sodium chloride, sodium acetate, ammonium acetate were purchased from Chempur (Piekary Śląskie, Poland), trisodium citrate was purchased from Avantor (Gliwice, Poland) and disodium citrate was purchased from Acros Organics (Morris Plains, NJ, US). Sorbents, silica functionalized with primary–secondary amine (PSA) and octadecyl groups were purchased from Agilent Technologies (Santa Clara, CA, USA). Certificated Reference Material BCR^®^ 646 was purchased from Merck (Darmstadt Germany). Ultrapure water was obtained from a Millipore water purification system (MiliQ, Billerica, MA, US) equipped with UV-lamp (resistivity of 18.2 MΩ.cm (at 25 °C) and a TOC value below 5 ppb).

### 3.2. Standard Solutions

The stock solutions of 1 mg/mL of TBT, TPhT, and TBT-d27 were prepared in methanol. The working standard solutions were prepared by dilution of the stock solution with appropriate amount of the methanol just prior the use. All stock solutions were stored at −25 °C.

### 3.3. Chromatographic Separation Conditions and Parameters of Mass Spectrometry

Instrumental analyses were performed using Agilent 1260 Infinity (Agilent Technologies, Santa Clara, CA, USA) equipped with a degasser, autosampler and binary pump, coupled to a Hybrid Triple Quadrupole/Linear Ion trap mass spectrometer (QTRAP^®^ 4000, AB SIEX, Framingham, MA, USA). The curtain gas, ion source gas 1, ion source gas 2 and collision gas (all high purity nitrogen) were set at 280 kPa, 380 kPa, 410 kPa and “high” instrument units, respectively. The ion spray voltage and source temperature were 5500 V and 600 °C, respectively. Kinetex RP–18 column (100 mm, 4.6 mm, particle size 2.6 µm) supplied by Phenomenex (Torrance, CA, USA) was used. The column temperature was 40 °C; eluent flow rate was 0.5 mL/min. The eluent was prepared from two solutions: A—0.2% formic acid in water and B—0.2% formic acid in acetonitrile. The concentration of solution B in eluent was 5% for 2 min, after that, the concentration increased to 95% in 7.5 min and for the next 5 min was 95%. The injection volume was 10 µL. The organotin compounds were analyzed in multiple reaction monitoring (MRM) mode. Two ion transitions (precursor→ product ion) for TBT and TPhT were presented in Table 5.

### 3.4. Sample Collection and Preparation

Sediments samples were collected from the Odra River Estuary by the use of Van Veen grab sampler. The volume of each collected sample was 3 liters. Samples were kept in 4 °C till arrival to the laboratory. Then were frozen in −80 °C, freeze-dried and stored in −80 °C till analysis. Before analysis the sediments were grounded in an agate mortar and sieved through 0.063 mm sieve. The blank samples of sediments used in extraction optimization and method validation were prepared by drying in a vacuum dryer for 1 h at 100 °C and 50 kPa in order to remove tributyltin and triphenyltin. After that, the TBT and TPhT residues were not found in the blank samples of bottom sediments.

### 3.5. Optimisation of the QuEChERS Extraction Procedure

The choice of optimal extraction parameters contained investigation of solvent composition, QuEChERS salts, extraction time and agitation technique as well as sorbent used for clean-up of the extract. Bottom sediment collected from the Odra River Estuary was taken for method optimization. Prior to use, blank samples were spiked with TBT and TPhT and kept in 4 °C for 24 h. The concentration of each organotin compound in sample after spiking was 2500 ng/g of solid. The effectiveness of the extraction from the sediments was compare to effectiveness of the extraction from MiliQ water solution based on (Equation (3)).

(3) $$Extraction\;recovery\;=\;A_{sample}/A_{miliQ}\;\times \;100\%,$$

where A_sample_ is peak area of spiked compounds extracted from sediment and A_miliQ_ is peak area of spiked compounds extracted from MiliQ water solution. It was assumed that OTC compounds were completely extracted from synthetic solution of analytes prepared in MiliQ water.

### 3.6. Extraction Procedure

Sample with about 0.125 g of sediment was placed in 2 mL Eppendorf^®^ tubes. For extraction optimization and method validation, the blank samples were spiked with TBT and TPhT and kept in 4 °C for 24 h. As an internal standard, 25 µL of deuterated tributyltin (TBT-d27) was added followed by 200 µL of MiliQ water. Then the samples were vigorously shaken by the use of vortex shaker for 1 min and 250 µL of 5% formic acid in acetonitrile was added. The samples were put in vessel with ice and 100 mg of ammonium acetate was added. Then they were extracted by ultrasonication for 5 min and shaken for 15 min (1500 rpm). The samples were centrifuged for 5 min (relative centrifuge force was 4472 g) and the supernatant was collected for farther analysis.

### 3.7. Method Validation

The method validation was performed according to the European Medicines Agency guideline. Briefly, the linearity range was selected as 1–4000 ng/g of sediment for TBT and 5–4000 ng/g of sediment for TPhT. Calibration curves were prepared in quadruplicate. Lower limit of quantification (LLOQ) was established as the concentration of TBT and TPhT, for which MS signal to noise ratio is equal or greater than 5, with precision below 20% and accuracy ±20%. A signal-to-noise ratio (S/N) of three was used to calculate the limit of detection (LOD). Repeatability (within-run precision) was estimated for 5 repetitions. Between-run precision was estimated for 15 repetitions. Accuracy and precision of the method was estimated for samples spiked with Quality Control (QC) concentrations equal 3 ng/g (QC1), 2500 ng/g (QC2), and 4000 ng/g (QC3) for TBT, and 15 ng/g (QC1) 2500 ng/g (QC2) and 4000 ng/g (QC3) for TPhT.

Selectivity of MS signal and carry-over of the analytical method was tested separately for tributyltin, triphenyltin and internal standard tributyltin d-27. Selectivity assessment was performed by using 6 different blank samples of sediments spiked with 1 ng/g TBT and 5 ng/g TPhT. Carry-over was assessed based on 6 blank sample solutions which were injected after injections of high concentrated (1000 ng/mL) standard solutions of TBT, TPhT, and TBT-d27.

Absolute matrix effect (ME_A_), relative matrix effect (ME_R_), and extraction recovery (RE) was studied on 10 sediments from the Odra River Estuary differing in the content of total organic carbon (TOC) from 0.1% to 11.8%. The characteristic of the sediments is presented in Appendix (Table A1). For ME_A_ and ME_R_ evaluation, the samples were spiked with TBT and TPhT to concentrations corresponding 2500 ng/g, 1000 ng/g, 500 ng/g, 100 ng/g, and 50 ng/g of solid. ME_A_ was calculated based on the slope of the calibration curve (y = ax + b) established by calibration solutions prepared in extracts of control sediments (a_control_) and the calibration curve established by calibration solutions prepared in MiliQ water (a_miliQ_) (Equation (4)).

(4) $$ME_{A}\;=\;a_{control}/a_{miliQ}\;\times \;100\%,$$

ME_R_ was calculated for 10 sediments as coefficient variation (CV%) of normalized ME_A_ referred as a ratio of absolute matrix effect of analyte (b_analyte_) to absolute matrix effect of internal standard (b_IS_) (Equation (5)).

(5) $$ME_{R}\;=\;CV\%\;(b_{analyte}/b_{IS}),$$

Additionally, for five samples the absolute matrix effect was also determined before and after sieving through 0.063 mm sieve. The extraction recovery was determined by comparing the peak areas of blank samples spiked with TBT and TPhT (1000 ng/g of solid) before and after extraction. Samples used for recovery and matrix effect were characterized by granulometry, pH of MiliQ water extract, conductivity of MiliQ water extract and total organic carbon (TOC), total nitrogen content (N), total hydrogen content (H), acid volatile sulfur (AVS), total phosphorus (P), sum of 16 priority polycyclic aromatic hydrocarbons (PAH), and sum of heavy metals (arsenic, barium, cadmium, cobalt, chromium, copper, iron, molybdenum, nickel, mercury, manganese, lead, tin, and zinc). Heavy metals were analysed by the use of inductively coupled plasma mass spectrometry ICP–MS. Elemental analysis (N, H, S, P, and heavy metals), TOC and PAH analyses were performed in Polish Geological Institute–National Research Institute according to accredited methods (in accordance with ISO-17025). Granulometric analysis was performed at University of Szczecin (in accordance with ISO 13320). Conductivity and pH of MiliQ water were measured in Medical University of Warsaw.

Stability of TBT and TPhT was evaluated in various conditions using blank samples of sediments spiked with TBT and TPhT to concentration equals 3 ng/g for TBT and 15 ng/g for TPhT (at low OTC concentrations) and 4000 ng/g for TBT and TPhT (at high OTC concentrations). The stability of analyte in the matrix was tested for three cycles of freeze and thaw (samples were frozen for at least 12 h after thawed). Short-term stability was tested for samples kept at room temperature for 4 h. Long-term stability was tested after storage of samples at −25 °C for 30 days. Stability of the extract in the autosampler was tested after 24 h and 48 h. Stability of working standards at concentration 10 µg/mL after storage at −25 °C for 30 days were also evaluated.

The certified reference material BCR 646 (European Commission Joint Research Centre Institute for Reference Materials and Measurements, Geel, Belgium) was used to validate the analytical method. The material consists of a dried and ground harbor bottom sediment sample with a particle size <90 µm and TBT and TPhT at concentration 480 and 29 ng/g, respectively. Validation of the method was based on results of six repetitions of OTC determination in BCR 646.

### 3.8. Real Sample Analysis

The concentration of tributyltin and triphenyltin in ten real samples collected from the Odra River Estuary were determined with use of the new validated method. Geographical localizations of sampling sites and depths of sampling are presented in Table 6.

### 3.9. Statistical Analysis

The statistical analysis of the results was performed with the STATISTICA version 13.1 for Windows (TIBCO Software Inc.; Palo Alto, CA, USA) and Metaboanalyst 4.0. Student′s t–Test was used for comparison of samples. Spearman′s correlation was used to measure the relationship between parameters of the analytical method and characteristic of sediment. Principal component analysis (PCA) was used to visualize the differences between sediment samples with high and low ME_A_ depending on their properties i.e.; pH, granulometry, elemental analysis, PAH.

## 4. Conclusions

The analytical method for the analysis of tributyltin and triphenyltin in sediments based on the combination QuEChERS extraction with LC–MS/MS technique was successfully developed and applied in pilot study TBT and TPhT pollution in bottom sediments of the Odra River Estuary, north-westernmost part of Poland. The method met acceptance all validation criteria according to guideline on bioanalytical method validation issued by European Medicines Agency. The determined concentrations of TBT and TPhT in reference material BCR 646 by the method is in good accordance with certificated concentrations. Additionally, the method is less time consuming, cheaper and more environmentally-friendly compared to widely used method ISO 23161. The time of sample preparation is significantly shorter due to simplicity of the method. The most important is also low use of organic reagents in QuEChERS extractions. In the new method only 0.25 mL of acetonitrile is needed, whereas ISO 23161 method requires 5–10 mL of hexane and 0.5–1 mL of highly volatile and toxic tetrahydrofuran. The use of high quantity of other substances and sodium tetraethylborate, as derivatization agent, is also needed by ISO method. Comparison of the different analytical methods is presented in Table A2

The studies have shown that clay minerals present in bottom sediments may affect negatively the sensitivity of the method, both directly and indirectly. Tributyltin and triphenyltin bound strongly to clay minerals. This may slightly decrease extraction efficiency. The indirect effect is that clay minerals accumulate large amounts of organic compounds. Those compounds may affect ionization of tin compounds in ESI source of the MS.

The results of preliminary environmental monitoring of organotin compounds indicated that the presence of those compounds is likely caused by activity of maritime industry. The presence of triphenyltin may be also caused by agriculture activity. Further studies are required to assess persistence of organotin compounds in the sediments of the Odra River Estuary. The presence of organotin compounds in the bottom sediments should be taken into consideration during environmental management of dredged bottom sediment.

## Figures and Tables

**Table 1:** The combinations of aqueous and organic solutions constituents used in the optimization procedure.

Composition Number	Aqueous Solution	Organic Solution
1	MiliQ water	Acetonitrile
2	30 mM KH_2_PO_4 _solution in water (pH = 7)	Acetonitrile
3	MiliQ water	Acetonitrile + 5% formic acid
4	30 mM KH_2_PO_4 _solution in water (pH = 7)	Acetonitrile + 5% formic acid

**Table 2:** Precision and accuracy data for tributyltin (TBT) and triphenyltin (TPhT).

**TBT**				
Nominal Concentration (ng/g)	1	3	2500	4000
Within-run precision (%) (*n* = 5)	1.5–2.7 ^a^	2.8–3.9	5.3–6.2	2.1–3.0
Within-run accuracy (%) (*n* = 5)	95–102 ^b^	85–104	98–106	98–101
Between-run precision (%) (*n* = 15)	1.9^ a^	3.5	5.6	2.6
Between-run accuracy (%) (*n* = 15)	102^ b^	99	103	100
**TPhT**				
Nominal concentration (ng/g)	5	15	2500	4000
Within-run precision (%) (*n* = 5)	2.2–6.6^ a^	3.2–9.3	4.0–7.6	3.7–9.5
Within-run accuracy (%) (*n* = 5)	108–117^ b^	91–97	97–102	100–104
Between-run precision (%) (*n* = 15)	4.3^ a^	6.8	5.7	5.7
Between-run accuracy (%) (*n* = 15)	112^ b^	95	101	102

Accepted precision: ≤15% (^a^ ≤ 20%). Accepted accuracy: 85–115% (^b^ 80–120%).

**Table 3:** Parameters of stability tests for tributyltin (TBT) and triphenyltin (TPhT) in sediments.

		TBT		TPhT	
		3 ng/g	4000 ng/g	15 ng/g	4000 ng/g
Long-term stability		114	113	113	112
Short-term stability		97	104	92	95
Freeze/thaw stability		114	104	90	91
Stability in autosampler	After 24 h	99	98	110	102
	After 48 h	100	99	112	105

Accepted precision: ≤15%. Accepted accuracy: 85–115%.

**Table 4:** Concentration of TBT and TPhT in below 0.063 mm fraction of bottom sediments, physicochemical characteristic of the sediments (conductivity and pH of MiliQ water extract, total organic carbon content (TOC), total nitrogen content (N), hydrogen content (H), acid volatile sulfur (AVS), phosphorus content (P), heavy metals (arsenic, barium, cadmium, cobalt, chromium, copper, iron, molybdenum, nickel, mercury, lead, tin, zinc), sum of 16 priority polycyclic aromatic hydrocarbons (PAH)) and grain size distribution.

Sediment Code	pH	Conductivity [mS]	TOC [%]	N [%]	H [%]	AVS [%]	P [%]	Heavy Metals [mg/kg]	PAH [µg/kg]	Sand [%] (0.063–1 mm)	Silt [%] (0.063–0.002 mm)	Clay [%] (<0.002 mm)	TBT [ng/g]	TPhT [ng/g]
S1	7.0	0.90	7.07	0.53	1.05	0.12	0.208	2399	9423	35	62	3	3296	<5
S2	6.9	1.13	6.53	0.6	1.16	0.4	0.302	2497	9828	27	70	3	3884	<5
S3	6.9	1.00	2.41	0.24	0.49	0.08	0.152	1213	2247	51	46	3	142	90
S4	6.7	0.95	8.81	0.66	1.24	0.35	0.297	2804	9625	30	67	3	1016	<5
S5	6.7	0.74	8.37	0.83	1.4	0.16	0.399	3123	no data	21	75	4	345	<5
S6	6.6	8.00	7.83	1.04	1.38	0.44	0.137	1920	2019.4	35	63	2	213	<5
S7	6.9	1.02	15.4	1.37	2.21	0.9	0.213	2814	73595	24	74	3	220	<5
S8	6.7	1.07	7.67	0.75	1.42	0.38	0.794	3318	no data	34	63	3	750	<5
S9	7.1	0.88	9.13	0.91	1.43	0.1	0.136	1704	no data	32	66	3	193	<5
S10	6.8	1.15	7.57	0.82	1.51	0.27	0.385	3710	no data	14	82	4	5263	9

**Table 5:** MS/MS optimized parameters: Declustering potential (DP), collision energy (CE), entrance potential (EP), and collision cell exit potential (CXP) for quantitative and qualitative product ions of examined compounds.

Compound	[M+H]+ Ion	Quantitative Product Ion	DP [V]	CE [V]	EP [V]	CXP [V]	Qualitative Product Ion	DP [V]	CE [V]	EP [V]
Tributyltin	291	179	71	19	19	12	122	71	10	33
Tributyltin deuterated	318	190	76	21	19	14	126	76	37	33
Triphenlyltin	351	196	126	10	37	14	119	126	10	41

**Table 6:** Geographic coordinates, depth of sampling and localization name of sediments collected from the Szczecin Lagoon.

Sample Number	Geographic Coordinates		Depth of Sampling Measured from Water Surface [m]	Localization
	Latitude–N	Longitude–E		
S1	53°27.328′	14°35.968′	2.2	Szczecin Shipyard
S2	53°27.336′	14°35.434′	2.5	Gunica River
S3	53°26.300′	14°35.280′	10.3	Elevator “Ewa”
S4	53°27.621′	14°36.102′	2.2	Swieta River
S5	53°27.068′	14°36.287′	2.1	Dabie Lake
S6	53°54.329′	14°15.249′	5.6	Piast Canal
S7	53°33.481′	14°34.578′	1.9	Larpia River– Police–
S8	53°24.227′	14°32.492′	2.0	Szczecin Harbor
S9	53°23.895′	14°37.698′	5.0	Szczecin – Dąbie Marina Club
S10	53°27.895′	14°35.923′	1.7	Szczecin– West Odra River

## Appendix A

**Table A1:** Localization, characteristic parameters (conductivity, pH, total organic carbon content (TOC), total nitrogen content (N), hydrogen content (H), acid volatile sulfur (AVS), phosphorus content (P), heavy metals (arsenic, barium, cadmium, cobalt, chromium, copper, iron, molybdenum, nickel, mercury, lead, tin, zinc), sum of 16 priority polycyclic aromatic hydrocarbon (PAH)) and granulometry (sand fraction (0.063–1 mm), silt fraction (<0.063–0.002 mm), clay fraction (<0.002 mm)) of 10 sediments used in matrix effect and recovery evaluation.

Geographic coordinates	Localisation	ME_A_ TBT [%]	ME_A_ TPhT [%]	RE TBT [%]	RE TPhT [%]	pH	Conductivity [mS]	TOC [%]	N [%]	H [%]	AVS [%]	P [mg/kg]	Heavy Metals [mg/kg]	PAH [µg/kg]	Sand [%]	Clay [%]	Silt [%]
N 53°46′881′′ E 14°35′224”	Skoszewska Bay	79	77	97	95	7.1	1.63	3.82	0.45	0.62	0.26	0.061	1808	1516	55	1	43
N 53°55.698′ E 014°45.278′	South part of Cicha Bay	80	63	92	88	7.2	1.92	11.8	1.34	1.57	0.61	0.094	1977	4460	38	1	61
N 53°51.209′ E 014°18.107′	Karsibor	86	86	98	94	7	5.01	1.48	0.41	0.32	0.06	0.036	404	622	65	1	34
N 53°45.638′ E 014°17.685′	Szczecin Lagoon	71	73	91	89	7.4	3.55	6.85	0.94	1.15	0.38	0.128	4183	4091	51	1	48
N 53°42.946′ E 014°21.396′	Warnolecka Bay	70	72	85	86	7.7	2.51	7.99	0.99	1.28	0.24	0.105	3477	6517	29	2	69
N 53°39.319′ E 014°36.367′	Roztoka Odrzanska	83	64	98	91	6.9	1.13	6.53	0.6	1.16	0.4	0.302	2497	6517	52	1	46
N 53°27.336′ E 014°35.434′	West Odra River	58	58	87	85	6.9	1.13	7.04	0.665	1.24	0.53	0.297	2594	9828	26	3	71
N 53°26.300′ E 014°35.280′	Elevator „Ewa”	74	76	88	91	6.9	1	2.87	0.26	0.52	0.08	0.168	1300	2247	49	3	48
N 54°00.598′ E 014°46.232′	Kamienski Lagoon	92	92	106	99	7.3	3.15	0.09	0.03	0.15	0	0.008	51	3926	97	2	1
N 53°35.917′ E 014°34.886′	Police	68	62	90	87	6.8	1.273	0.13	0.03	0.14	0.03	0.011	268	4924	96	1	3

**Table A2:** Comparison of analytical methods of tributyltin and triphenyltin determination.

Extraction Methods	Extraction Solvents	Derivatization	Instrument	LOD for TBT [ng/g]	LOD for TPhT [ng/g]	Ref.
QuEChERS	acetonitrile, H_2_O, formic acid	No	LC–MS/MS	0.6	2.4	This work
ASE	Methanol, H_2_O acetic acid and tropolone	NaBT_4_	GC–FPD	19	14	[21]
SPE	Tetrahydrofurane, hydrochloric acid, tropolone, hexane	Grignard′s reagent	GC–MS/MS	0.4–1.5	–	[45]
SPME	Hydrochloric acid, methanol	NaBT_4_	GC–FPD	1.7	20	[46]
MAE	Acetic acid, tartaric acid, iso-octane	NaBT_4_	GC–MS	126	–	[47]

ASE—Accelerated Solvent Extraction; GC-FPD—Gas Chromatography with Flame Photometric Detector; SPE—Solid Phase Extraction; SPME—Solid Phase Microextraction; MAE—Microwace Assisted Extraction, LOD—Limit of Detection calculated based on signal-to-noise ratio (S/N) of three.
